# Our New York Letter

**Published:** 1888-07

**Authors:** 


### Correspondence.

### OUR NEW YORK LETTER.

EIGHTY-FIVE THOUSAND DOLLARS FOR A TRAINING SCHOOL FOR MALE NURSES—THE RELATION BETWEEN PLEURISY AND CONSUMPTION—CHANGES IN THE POST-GRADUATE SCHOOL—ELECTRICITY VERSUS HANGING.

*To Editors Atlanta Medical and Surgical Journal:*

In a short time New York is to have a training school for male nurses. Mr. D. O. Mills, of this city, has given $85,000 for the purpose of erecting and completely outfitting such a school, on the Bellevue Hospital grounds, to be carried on in connection with the hospital. This will, I believe, be the first institution of this kind in the country. The Bellevue Training School for Nurses was started about fourteen years ago, and since then similar schools have sprung up all over the land, and hundreds of young women have graduated from them. Still, there has been a growing want felt for competent male nurses, and in many cases of sickness a male attendant is especially desirable.

When Mr. Mills' attention was called to the subject, he realized that there was a great field in this department open to men, and with a spirit of true philanthropy, offered to build an institution where the necessary instruction could be given to those anxious to learn this profession. The building, which is of brick and five stories high, will accommodate sixty nurses. It will be completely equipped with all that is necessary, and is to be under the charge of the Commissioners of Charities and Correction. Each applicant will be obliged to agree to stay in the institution for two years, at the end of which time, if found competent, he will receive a diploma.

During the course of instruction the nurses will receive their board free, and in addition small wages will be paid them. I have heard that the training schools of New York have more applicants than they can accommodate, and that those who apply are seldom residents of this city, but come from all over the United States.

A very interesting discussion took place a few nights ago at the Academy of Medicine on the relations between pleurisy and phthisis. Dr. B. F. Westbrook, in a paper presented, stated that there were cases in which the occurrence of pleurisy, although not tubercular in character, was soon followed by tuberculosis of the lungs. Here he regarded the pleurisy as an indication of a constitutional weakness, which led to the occurrence of the phthisis sooner or later. Pleurisy, he believed, might be tubercular in origin. In other cases, sero-fibrinous pleurisy was followed by fibroid phthisis. Pleurisy also might end in thorough recovery, but some months later signs of tuberculosis appeared at the apices of the lungs. Often in elderly persons chronic serous pleurisy finally developed tuberculosis. He said that after apparent recovery from pleurisy great care should be taken to completely restore the patient's health. With a family history of phthisis, the case should be watched for at least a year. In elderly persons he would not allow an effusion to remain over three weeks without aspirating. Respiratory gymnastics and the pneumatic cabinet were often beneficial in many cases.

Dr. J. Blake White thought it was a well-established fact that pleurisy often gave rise to tuberculosis in previously healthy persons. The prevention and curability of phthisis depended largely on increasing lung capacity, especially at the apices, as here activity is low. In fibroid pleurisy, blisters, exercises and hygienic measures should be employed for a long time. Dr. White believed that the bacillus tuberculosis was the result and not the cause of phthisis.

Dr. W. B. Wood said that in children the first evidence of a tendency to phthisis was feeble breathing power, which was principally caused by obstructed nasal respiration and thick, immovable pleura at the base of the lungs. Hence, treatment should be directed to the nose and free nasal respiration secured. Lung gymnastics should also be employed.

Dr. Brun thought more stress should be laid on the tubercular origin of pleurisy. Supposed primary pleurisy, he said, was often tubercular, as he had proved by autopsies, which showed tubercular changes in the pleura, while the lungs were normal. He believed this process could undergo resolution in the pleura or lung, just as it did in the peritoneum. As he had not derived much good from aspirating where absorption was delayed, he believed in waiting as long as possible.

Dr. Shattuck, of Boston, who was present, was also inclined to regard many cases of pleurisy as tubercular. Tuberculosis, he said, might exist, independent of any lung lesion, as a local and almost harmless condition. In the lungs the bacillus found the most favorable conditions for development. The difficulty of finding these germs in pleuritic effusion was no proof, he said, of their absence. Dr. Shattuck thought the best pulmonary gymnastics were obtained by mountain climbing.

Dr. E. G. Janeway said that undoubtedly tuberculosis often preceded what we thought to be primary pleurisy. Autopsies of pleurisy cases generally revealed tuberculosis, although during life there had been the ordinary history of inflammation of the pleura only. Rheumatic patients might, however, he believed, have pleurisy and get over it without any signs of tubercular trouble. When repeated tappings failed, he had seen the following mode of treatment succeed in getting rid of the effusion. The quantity of liquid drank should be less than a third or half of the urine voided. Salt and iodide of potassium should be administered at the same time, which, Dr. Janeway said, made the blood "thirsty" for fluid. The meeting was very largely attended, probably owing to the interesting and important subject under discussion.

The Post-Graduate School of this city has been making some valuable additions to their faculty. At a recent meeting Dr. A. Jacobi was made Professor of Diseases of Children. The other appointments were as follows: Dr. R. F. Weir, Professor of Clinical Surgery; Dr. P. A. Callan, Professor of Diseases of the Eye; Dr. L. B. Bangs, Professor of Genito-urinary and Venereal Diseases; Dr. O. B. Douglass, Professor of Diseases of the Nose and Throat.

Dr. W. A. Hammond has resigned the chair of Nervous Diseases. He was one of the founders of the institution.

Executing criminals by hanging is soon to be abolished here. The Governor has signed the bill and the law is that there shall be no hanging after January 1 next in New York. From this date electricity will take the place of the gallows. A. F.

*June 8, 1888.*

---

### THE MEDICAL LAWS OF ALABAMA.

---

*Editors Atlanta Medical and Surgical Journal:*

